# Complete mitochondrial genome sequence of the giant mud worm *Paraleonnates uschakovi* Khlebovich & Wu, 1962 (Polychaeta: Nereididae)

**DOI:** 10.1080/23802359.2016.1214552

**Published:** 2016-11-22

**Authors:** Taeseo Park, Sang-Hwa Lee, Won Kim

**Affiliations:** a Animal Resources Division, National Institute of Biological Resources, Incheon, Korea;; b School of Biological Sciences, Seoul National University, Seoul, Korea;; c Graduate Program in Cellular Biology and Genetics, Colleage of Medicine, Chungbuk National University, Cheongju, Korea

**Keywords:** *Paraleonnates uschakovi*, complete mitogenome, Nereididae

## Abstract

Complete mitochondrial genome sequence of the giant mud worm *Paraleonnates uschakovi* (Polychaeta: Nereididae) was determined in this study for the first time. The mitogenome of *P. uschakovi* is 15,540 bp in length. It has 13 protein-coding genes, two rRNA genes, 22 tRNA genes and a non-coding region. Mitogenome analysis of *P. uschakovi* showed inversion in the positions of three tRNAs compared to the mitogenome sequences of *Perinereis aibuhitensis*, *P. nuntia* and *Platynereis dumerilii*. The phylogenetic position of *P. uschakovi* compared to 15 selected polychaetes was investigated. *P. uschakovi* was grouped into the family of Nereididae. It is closely related to the clade containing *Tylorrhynchus heterochaetus* and *Namalycastis abiuma*.

The genus *Paraleonnates* (Khlebovich & Wu 1962) belongs to family Nereididae (Blainville 1818). It is comprised of three valid species: *P. tenuipalpa* (Pflugfelder 1933), *P. uschakovi* (Khlebovich & Wu 1962), and *P. bolus* (Hutchings & Reid 1991). Of the three species, *P. uschakovi* is widely distributed at Korean and Chinese coasts, and Thailand coast of Andaman Sea (Paik 1977; Wu et al. 1985; Hong et al. 2012). This species is known as a fishing bait for mullet in Korea. It is also a source of alkaline protease (Joo et al. 2001). Molecular phylogenetic studies have been conducted to solve the taxonomic problem of cryptic nereidid species using mitochondrial partial genes such as COI and 16S (Park & Kim 2007; Tosuji & Sato 2008; Glasby et al. 2013). Complete mitochondrial genome sequence can provide more valuable evolutionary information compared to partial genes (Chen et al. 2016). So far, complete mitogenomes of five nereidid species have been determined. For future molecular evolutionary studies of nereidid worms, more complete mitogenome information is needed. The objective of this study was to determine the complete mitogenome sequence of *P. uschakovi.*

The specimen was collected in muddy tidal flat of Ganghwa Island, Republic of Korea. Voucher specimen was deposited at National Institute of Biological Resources (NIBRIV0000539555).

A single live specimen of *P. uschakovi* was used to obtain pure mitochondrial genomic DNA. Total genomic DNA extraction, sequencing, and gene annotation methods described by Song et al. (2016) were used. Phylogenetic tree was constructed using MEGA6 (Tamura et al. 2013).

The complete mitogenome of *P. uschakovi* was 15,540 bp in length (GenBank accession no. KX462988), containing a total of 37 genes (13 protein-coding genes, two rRNA genes, and 22 tRNA genes) and a non-coding region of about 1kb. Gene order of *P. uschakovi* is identical to that of the following two nereidid species: *Tylorrhynchus heterochaetus* (Quatrefages 1866) and *Namalycastis abiuma* (Grube 1872). However, tRNA-Met, tRNA-Asp, ATP8, and tRNA-Tyr showed inversions in the other three nereidid species: *Perinereis aibuhitensis* (Grube 1878), *P. nuntia* (Lamarck 1818), and *Platynereis dumerilii* (Audouin & Milne Edwards 1834). Thus, nereidid species are distinguishable with two different gene order groups so far. The gene order group including *P. uschakovi* has similar polychaetes ground pattern (tRNA-Asp, ATP8, tRNA-Tyr). The initiation codons of genes include ATT (cox1, cytb, nad2, nad5, nad6), ATA (cox2, nad3, nad4L), ATG (cox3, nad4, atp6, atp8), and ATC (nad1). Stop codons include TAG (cox1, nad6) and TAA (cox2, nad2, nad3, nad4, nad4L, atp6) excepting some genes (cox3, cytb, nad1, nad5, atp8) terminated with T.

To examine the phylogenetic position of *P. uschakovi*, maximum-likelihood analysis was performed using concatenated protein-coding genes from 15 selected polychaetes. The resulting tree showed that *P. uschakovi* was grouped into family Nereididae. It is closely related to the clade containing *T. heterochaetus* and *N. abiuma* with high bootstrap value (Figure 1).

**Figure 1.**
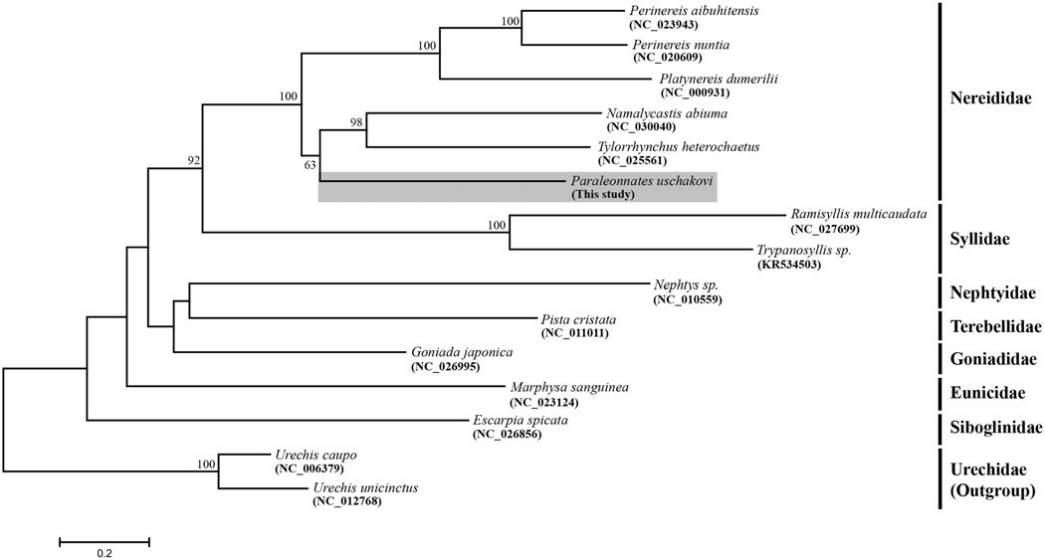
Maximum-likelihood (ML) tree based on 15 mitogenome sequences including *Paraleonnates uschakovi* (present study). It was constructed using MEGA 6.0 software. Bootstrap replicates were performed 1000 times. Bootstrap values above 60% were indicated on the cladogram.

The complete mitogenome information of *P. uschakovi* determined from this study will be useful for detailed phylogenetic and evolutionary analyses among nereidid species in the future.
